# London Lancet

**Published:** 1861-07

**Authors:** 


THE LONDON LANCET for June, from whose richly-stored pages we re-published, in our June number, the valuable *Directions to Army Surgeons on the Field of Battle*, contains the Index for the half-yearly volume, which it completes. COOTE takes up the usually neglected subject of *Curvatures of the Spine*, in his Clinical Lectures, as also *Knock-Knee* (*genu valgum*); *Bow-Leg* (*genu extrorsum*); etc., and the number is, as usual, replete with original papers, hospital reports, clinical records, correspondence, etc. A complete alphabetical Index for the ten years between 1851 and 1860, will be published on the 15th inst.

